# A proposal for the classification of early complications during percutaneous central venous access: previous note

**DOI:** 10.1590/1677-5449.202501472

**Published:** 2026-09-14

**Authors:** José da Cruz Moura Campêlo, Luís Gustavo Cavalcante Reinaldo, Alexandre da Costa Machado Matos Terceiro, Ocilio Ribeiro Gonçalves

**Affiliations:** 1 Universidade Federal do Piauí – UFPI, Teresina, PI, Brasil.; 2 Universidade Federal do Norte do Tocantins – UFNT, Araguaína, TO, Brasil.

**Keywords:** central venous catheterization, complications, classification, patient safety

## Abstract

Central venous access is a common hospital procedure that plays an essential role in many clinical situations. Despite its widespread use, there is no standardized classification for early complications, which limits systematic analysis and development of health indicators. This study aimed to propose an objective and applicable classification for early complications of central venous access, encompassing events related to puncture and catheter positioning. This was a longitudinal observational study conducted at the Universidade Federal do Piauí teaching hospital (HU-UFPI) from October 2024 to April 2025, enrolling patients undergoing central venous access placement by the Clinical Surgery Unit team. Clinical and procedural variables were assessed, including ultrasound guidance, puncture site, catheter positioning, and post-procedure complications. The proposed classification groups complications into five categories: I, IIA, IIB, IIIA, and IIIB. In the sample (n = 35), 85.7% of observed complications were classified as category I, 11.4% as IIA, and 2,9% as IIB, with no major complications observed. The proposed classification appears to be potentially useful for standardizing the description of early complications, although the findings should be interpreted with caution due to the small sample size, absence of major complications, and single-center design.

## INTRODUCTION

Central venous access is a widely performed procedure in hospital settings and is an essential part of patient management in many clinical situations.^1^ Modern percutaneous techniques were based on the Seldinger technique and classical descriptions of subclavian venous puncture.^2-4^ Despite its widespread use and importance for provision of care, central venous catheterization is not risk free and can involve complications with potential impact on morbidity and mortality. These complications can occur during the procedure or over the first few days afterwards and can be related to the puncture or to catheter positioning.

While central venous catheterization is a routine procedure in several different hospital sectors, there is an important gap in the literature: there is no standardized classification for immediate and early complications related to these procedures. Brazilian and international recommendations stress the importance of ultrasound guidance of vascular access^5,6^ and review studies have analyzed its impact on the safety of central venous catheterization.^7,8^ Moreover, technical aspects and complications related to the puncture site are widely described in the literature.^9-12^ The lack of a classification limits systematic analysis of interventions, compilation of health care indicators, and comparisons of different centers, techniques, and devices. A systematic classification could contribute to standardization of communication between professionals, to documentation in medical records, and to development of evidence-based institutional protocols.

Against this background, the objective of this study was to propose an objective classification applicable to early complications of central venous access, encompassing both complications related to puncture and those linked to catheter positioning.

## METHODS

This is a longitudinal, observational, cohort study conducted at a teaching hospital run by the Universidade Federal do Piauí, in the Brazilian city of Teresina, Piauí, from October 2024 to April 2025. Patients were enrolled who had central venous access prepared by the HU-UFPI Clinical Surgery Unit team, using double-lumen and totally implantable catheters. Patients were excluded if their access had not been fitted by the Clinical Surgery Unit team or if they had had a peripherally inserted central catheter fitted by the clinical nursing team or had undergone any other type of central catheterization not included in the inclusion criteria. Patients were followed-up for the first 48 hours after the procedure to identify early complications.

Variables were analyzed related to age, sex, indication for central venous access, use of ultrasound to guide puncture, puncture site, type of catheter, position on simple chest X-ray, and occurrence of post-procedural complications. Based on the collected data and on analysis of the observed complications, a conceptual-operational classification was developed for early complications of central venous access, termed the Campelo et al. classification. Table 1 summarizes the proposed classification. This proposal is neither an exhaustive checklist nor a validated diagnostic instrument, since the primary objective is to standardize description and stratification of early complications observed in clinical practice. Figure 1 shows a flowchart illustrating the process of inclusion of access procedures in the study.

**Table 1:** Table summarizing the Campelo et al. classification for early complications of central venous access.

**Category**	**Description**
I	Catheter correctly positioned, no apparent complications.
	This category is essential, because it constitutes the ideal scenario in which the catheter is correctly positioned and there are no catheter-related complications. Correct positioning of the catheter should be confirmed by imaging exams, such as chest X-ray.
IIA	Minor complication related to puncture, with no need for intervention, or managed conservatively.
	Covers minor events caused by puncture, without significant clinical repercussions, such as local hematoma, inadvertent arterial puncture without repercussions, and multiple attempts at the same site, not requiring a change of site. These complications are monitored clinically and do not need surgical intervention or invasive procedures.
IIB	Minor complication related to catheter positioning, without compromising catheter function. These are minor positioning errors that do not compromise the function of the device, such as malpositioning of the distal tip with no clinical repercussions (for example, catheter tip in the internal jugular vein), which may require elective repositioning.
IIIA	Major complication related to puncture, requiring intervention.
	These are severe events caused by puncture that require immediate intervention, such as pneumothorax, hemothorax, expanding hematoma, or arterial injury, with potential impact on morbidity and mortality.
IIIB	Major complication related to catheter positioning, requiring intervention.
	These are serious situations related to malpositioning that require immediate intervention, such as vascular or cardiac perforation, tamponade, arrhythmia due to intracardiac positioning of the tip, or incorrect path with clinical repercussions.

Source: Prepared by the authors. Note: This is a conceptual and operational classification and is not an exhaustive checklist or a validated diagnostic instrument.

**Figure 1:**
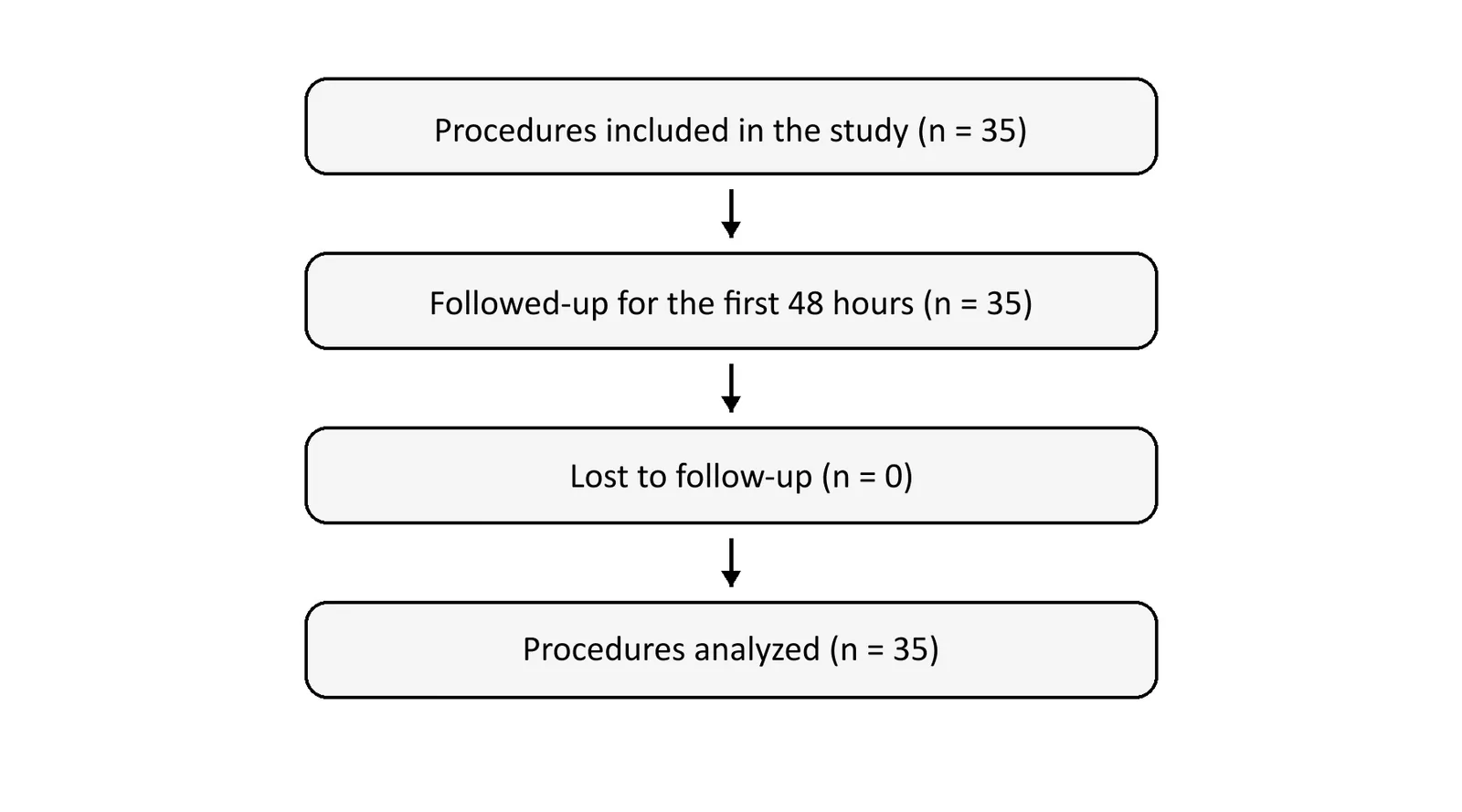
Flow diagram of inclusion of procedures in the study. Notes: The figure illustrates the flow of the procedures included in the final study sample (n = 35), with 48-hour follow-up and no losses to follow-up. The objective is to document follow-up and analysis of the sample.

This study was approved by the Research Ethics Committee at the Hospital Universitário, Universidade Federal do Piauí, under Substantiated opinion number 7.133.140 and CAAE submission number 83499724.6.0000.8050.

## RESULTS

The study sample comprised 35 patients who had central venous access fitted by the HU-UFPI Clinical Surgery Unit team, from October 2024 to April 2025. Mean patient age was 56.7 ± 13.8 years, with a predominance of female patients (60.0%). The most common indication for central venous access was inability to use peripheral access (51.4%), followed by chemotherapy (17.1%), major surgery (14.3%), total parenteral nutrition (14.3%), and administration of vasoactive drugs (2.9%). The right subclavian vein was the most common puncture site (40.0%), followed by the right internal jugular vein (37.1%). Ultrasound was used to guide puncture in 40.0% of the procedures and double lumen catheters were the most common type employed (82.9%). Table 2 lists sociodemographic data and clinical characteristics for the sample.

**Table 2:** Sociodemographic and clinical characteristics of the sample (n = 35).

**Variable**	**Category**	**n (%)**
Sex	Female	21 (60.0%)
	Male	14 (40.0%)
Age	Mean ± SD	56.7 ± 13.8 years
Indication for venous access	Impossible to use peripheral access	18 (51.4%)
	Chemotherapy	6 (17.1%)
	Major surgery	5 (14.3%)
	Total parenteral nutrition	5 (14.3%)
	Vasoactive drugs	1 (2.9%)
Puncture site	Right subclavian vein	14 (40.0%)
	Right internal jugular vein	13 (37.1%)
	Left internal jugular vein	4 (11.4%)
	Left subclavian vein	2 (5.7%)
	Right femoral vein	1 (2.9%)
	Multiple sites	1 (2.9%)
Ultrasound used	No	21 (60.0%)
	Yes	14 (40.0%)
Type of catheter	Double lumen	29 (82.9%)
	Totally implantable	6 (17.1%)

SD = standard deviation.Values expressed as absolute numbers and percentages, except where indicated otherwise.

The Campelo et al. classification distributes complications into five categories: I (no complications), IIA (minor complications related to puncture), IIB (minor complications related to positioning), IIIA (major complications related to puncture), and IIIB (major complications related to positioning). Category I was the most frequent in the study sample, accounting for 85.7% of cases. Minor complications related to puncture occurred in 11.4% of procedures, while minor complications related to positioning were observed in 2.9% of cases. No major complications were recorded in the study sample.

There were four cases of minor complications related to puncture, including local hematoma, arterial puncture with no clinical repercussions, and multiple attempts at the same site, with no need to change site. Among the minor complications related to positioning, there was one case of a malpositioned catheter that did not compromise its function, with the distal tip in the internal jugular vein.

## DISCUSSION

This study proposes an objective and applicable classification for early complications of central venous access, organized in five categories and encompassing events related to puncture and to catheter positioning. The predominance of procedures with no complications and the absence of major complications suggests a favorable safety profile in the setting analyzed, although this finding should be interpreted with care, considering the proposal’s pilot nature and the small sample size.

The proposal’s main contribution is its simplicity and practical applicability. By standardizing communication about complications of central venous access, the classification can facilitate documentation in medical records, comparisons between centers, and compilation of health care indicators. Additionally, it covers both events related to puncture and those related to positioning, offering a more comprehensive view of the procedure. It is a conceptual and operational model and is not intended to serve as an exhaustive checklist or as a validated diagnostic instrument.

In the Brazilian context, the Brazilian Society of Angiology and Vascular Surgery vascular access guidelines stress the role of ultrasound for puncture guidance, careful choice of site of access, and radiological confirmation of positioning as measures associated with reduced complications.^13^ The proposed classification is aligned with these recommendations, facilitating its incorporation into Brazilian health care practice, to the extent that it standardizes description of events — related to puncture and positioning — that the guidelines seek to prevent, contributing to compilation of health care indicators that are comparable between different Brazilian centers.

The limitations of the study include the small sample size, the single center design, the absence of major complications, and the lack of interobserver reproducibility assessment. These factors limit generalization of the findings and indicate a need for multicenter studies with larger samples to conduct external validation of the proposal.

## CONCLUSIONS

The proposed classification is a pilot instrument for the stratification of early complications associated with central venous access, with the potential to contribute to standardization in the description of these events and to improved communication between health professionals. However, the evidence presented in this study is limited, particularly because of the small number of participants, the single-center design, and the absence of major complications in the study sample. Therefore, the applicability and clinical impact of the classification still require confirmation in future studies, ideally with multicenter designs, larger samples, and formal assessment of interobserver reproducibility.

## DISPONIBILIDADE DE DADOS

Todos os dados gerados ou analisados estão incluídos neste artigo e/ou no material suplementar.

## References

[1] Di Santo MK, Takemoto D, Nascimento RG (2017). Cateteres venosos centrais de inserção periférica: alternativa ou primeira escolha em acesso vascular?. J Vasc Bras.

[2] Seldinger SI (1953). Catheter replacement of the needle in percutaneous arteriography; a new technique. Acta Radiol.

[3] Aubaniac R (1952). L’injection intraveineuse sous-claviculaire: advantages et technique. Presse Med.

[4] Yoffa D (1965). Supraclavicular subclavian venepuncture and catheterisation. Lancet.

[5] Franco-Sadud R, Schnobrich D, Mathews BK (2019). Recommendations on the use of ultrasound guidance for central and peripheral vascular access in adults: a position statement of the Society of Hospital Medicine. J Hosp Med.

[6] Lamperti M, Bodenham AR, Pittiruti M (2012). International evidence-based recommendations on ultrasound-guided vascular access. Intensive Care Med.

[7] Hind D, Calvert N, McWilliams R (2003). Ultrasonic locating devices for central venous cannulation: meta-analysis. BMJ.

[8] Lalu MM, Fayad A, Ahmed O (2015). Ultrasound-guided subclavian vein catheterization: a systematic review and meta-analysis. Crit Care Med.

[9] Sebastião A (2010). Acessos venosos centrais e arteriais periféricos – aspectos técnicos e práticos. Revista Brasileira de Medicina Intens..

[10] Parienti JJ, Mongardon N, Mégarbane B (2015). Intravascular complications of central venous catheterization by insertion site. N Engl J Med.

[11] McGee DC, Gould MK (2003). Preventing complications of central venous catheterization. N Engl J Med.

[12] Merrer J, De Jonghe B, Golliot F (2001). Complications of femoral and subclavian venous catheterization in critically ill patients: a randomized controlled trial. JAMA.

[13] Harduin LO, Barroso TA, Guerra JB (2023). Diretrizes sobre acesso vascular para hemodiálise da Sociedade Brasileira de Angiologia e de Cirurgia Vascular. J Vasc Bras.

