# Supplementary material for: A proposal for the classification of early complications during percutaneous central venous access: previous note
Source: J Vasc Bras. 2026 Sep 14;25:e20250147. doi: 10.1590/1677-5449.202501472 (PMC13671018; doi:10.1590/1677-5449.202501472)
Supplement: Translation

# Translation: Proposta de classificação para complicações precoces nos procedimentos para acesso venoso central por punção: nota prévia

**DOI:** 10.1590/1677-5449.202501471

**Authors:** José da Cruz Moura Campêlo, Luís Gustavo Cavalcante Reinaldo, Alexandre da Costa Machado Matos Terceiro, Ocilio Ribeiro Gonçalves

**Affiliations:** 1 Universidade Federal do Piauí – UFPI, Teresina, PI, Brasil.; 2 Universidade Federal do Norte do Tocantins – UFNT, Araguaína, TO, Brasil.

**Keywords:** cateterismo venoso central, complicações, classificação, segurança do paciente

## Abstract

O acesso venoso central é um procedimento frequente no ambiente hospitalar e essencial em diversas situações clínicas. Apesar de sua ampla utilização, não existe uma classificação padronizada para complicações precoces, o que dificulta análises sistemáticas e a criação de indicadores em saúde. Este estudo teve como objetivo propor uma classificação objetiva e aplicável para complicações precoces de acessos venosos centrais, abrangendo eventos relacionados à punção e ao posicionamento do cateter. Trata-se de estudo observacional longitudinal, realizado no Hospital Universitário da Universidade Federal do Piauí (HU-UFPI) entre outubro de 2024 e abril de 2025, com inclusão de pacientes submetidos a acesso venoso central pela equipe da Unidade de Clínica Cirúrgica. Foram avaliadas variáveis clínicas e procedimentais, incluindo uso de ultrassonografia, sítio de punção, posicionamento do cateter e complicações pós-procedimento. A classificação proposta distribui as complicações em cinco categorias: I, IIA, IIB, IIIA e IIIB. Na amostra (n = 35), 85,7% foram classificados como categoria I, 11,4% como IIA e 2,9% como IIB, sem registro de complicações maiores. A classificação proposta mostrou-se potencialmente útil para padronizar a descrição das complicações precoces, embora os achados devam ser interpretados com cautela em razão do tamanho reduzido da amostra, da ausência de complicações maiores e do caráter unicêntrico do estudo.

## INTRODUÇÃO

Os acessos venosos centrais constituem procedimentos amplamente realizados no ambiente hospitalar, sendo fundamentais para o manejo de pacientes em diversas situações clínicas^1^. As técnicas percutâneas modernas foram consolidadas a partir da técnica de Seldinger e de descrições clássicas de punção venosa subclávia^2-4^. Apesar de sua ampla utilização e importância assistencial, o cateterismo venoso central não é isento de riscos, podendo apresentar complicações com potencial impacto na morbimortalidade. Essas complicações podem ocorrer durante o procedimento ou nos primeiros dias após sua realização, estando relacionadas tanto à punção quanto ao posicionamento do cateter.

Embora os cateterismos venosos centrais sejam realizados rotineiramente em diferentes serviços, uma lacuna importante na literatura permanece: a ausência de uma classificação padronizada para as complicações imediatas e precoces desses procedimentos. Recomendações nacionais e internacionais destacam a importância da ultrassonografia na orientação de acessos vasculares^5,6^, e estudos de revisão também avaliaram seu impacto na segurança do cateterismo venoso central^7,8^. Além disso, aspectos técnicos e complicações relacionadas ao local de punção são amplamente descritos na literatura^9-12^. Essa carência dificulta a análise sistemática das intervenções, a elaboração de indicadores em saúde e a comparação entre diferentes serviços, técnicas e dispositivos. Uma classificação sistematizada poderia contribuir para a padronização da comunicação entre profissionais, para a documentação em prontuários e para o desenvolvimento de protocolos institucionais baseados em evidências.

Neste contexto, o presente estudo teve como objetivo propor uma classificação objetiva e aplicável para as complicações precoces dos acessos venosos centrais, abrangendo tanto aquelas relacionadas à punção quanto as vinculadas ao posicionamento do cateter.

## MÉTODOS

Trata-se de um estudo observacional longitudinal do tipo coorte, realizado no Hospital Universitário da Universidade Federal do Piauí (HU-UFPI), na cidade de Teresina, Piauí, no período de outubro de 2024 a abril de 2025. Foram incluídos pacientes submetidos a acessos venosos centrais pela equipe da Unidade de Clínica Cirúrgica do HU-UFPI, dos tipos cateteres de duplo lúmen e totalmente implantáveis. Foram excluídos os pacientes cujos acessos não foram realizados pela equipe da unidade, além daqueles submetidos a implante de cateter central de inserção periférica realizado pela equipe da enfermagem clínica e demais tipos de cateterismo central não previstos nos critérios de inclusão. Os pacientes foram acompanhados durante as primeiras 48 horas após o procedimento para identificação de complicações precoces.

Foram analisadas variáveis relacionadas à idade, ao sexo, à indicação do acesso venoso central, ao uso de ultrassonografia para guiar a punção, ao local de punção, ao tipo de cateter, ao posicionamento em radiografia simples de tórax de controle e à ocorrência de complicações pós-procedimento. Com base nos dados coletados e na análise das complicações observadas, foi desenvolvida uma classificação de caráter conceitual e operacional para as complicações precoces dos acessos venosos centrais, denominada Classificação de Campelo et al. A tabela-resumo da classificação proposta está apresentada na Tabela 1. Essa proposta não se configura como lista de verificação exaustiva nem como instrumento diagnóstico validado, tendo como objetivo principal padronizar a descrição e a estratificação das complicações precoces observadas na prática clínica. O fluxo dos procedimentos incluídos no estudo está apresentado na Figura 1.

**Tabela 1:** Tabela-resumo da Classificação de Campelo et al. para complicações precoces em acessos venosos centrais.

**Categoria**	**Descrição**
I	Cateter corretamente posicionado sem complicações aparentes.
	Esta categoria é fundamental, pois representa o cenário ideal em que o cateter está corretamente posicionado e não há complicações associadas. O correto posicionamento do cateter deve ser verificado por exames de imagem, como radiografia de tórax.
IIA	Complicação menor relacionada à punção, sem necessidade de intervenção ou com manejo conservador.
	Engloba eventos menores decorrentes da punção, sem repercussão clínica significativa, como hematoma local, punção arterial inadvertida sem repercussão e múltiplas tentativas no mesmo sítio sem necessidade de mudança de local. São monitorados clinicamente, dispensando intervenção cirúrgica ou procedimentos invasivos.
IIB	Complicação menor relacionada ao posicionamento do cateter, sem comprometimento de sua função.
	Refere-se a alterações menores do posicionamento que não comprometem a função do dispositivo, como mau posicionamento da extremidade distal sem repercussão clínica (por exemplo, ponta do cateter em veia jugular interna), podendo demandar reposicionamento eletivo.
IIIA	Complicação maior relacionada à punção, com necessidade de intervenção.
	Compreende eventos graves decorrentes da punção que exigem intervenção imediata, como pneumotórax, hemotórax, hematoma em expansão ou lesão arterial, com potencial impacto na morbimortalidade.
IIIB	Complicação maior relacionada ao posicionamento do cateter, com necessidade de intervenção.
	Abrange situações graves associadas ao mau posicionamento que requerem intervenção imediata, como perfuração vascular ou cardíaca, tamponamento, arritmias por posicionamento intracardíaco da extremidade ou trajeto aberrante com repercussão clínica.

Fonte: Elaborada pelos autores. Notas: Classificação de caráter conceitual e operacional, não configurando *checklist* exaustivo nem instrumento diagnóstico validado.

**Figura 1:** Fluxograma dos procedimentos incluídos no estudo. Notas: A figura descreve o fluxo dos procedimentos incluídos na amostra final do estudo (n = 35), com acompanhamento por 48 horas e ausência de perdas de seguimento. Portanto, seu objetivo é documentar o seguimento e a análise da amostra incluída.

O estudo foi aprovado pelo Comitê de Ética em Pesquisa do Hospital Universitário da Universidade Federal do Piauí (HU-UFPI), sob Parecer Consubstanciado nº 7.133.140 e CAAE 83499724.6.0000.8050.

## RESULTADOS

A amostra do estudo foi composta por 35 pacientes submetidos a acessos venosos centrais pela equipe da Unidade de Clínica Cirúrgica do HU-UFPI, durante o período de outubro de 2024 a abril de 2025. A idade média dos pacientes foi de 56,7 ± 13,8 anos, com predominância do sexo feminino (60,0%). Em relação à indicação do acesso venoso central, a impossibilidade de acesso periférico foi a mais frequente (51,4%), seguida pela necessidade de quimioterapia (17,1%), cirurgia de grande porte (14,3%), nutrição parenteral total (14,3%) e administração de drogas vasoativas (2,9%). Quanto ao local de punção, a veia subclávia direita foi a mais utilizada (40,0%), seguida pela veia jugular interna direita (37,1%). O uso de ultrassonografia para guiar a punção foi observado em 40,0% dos procedimentos, e os cateteres de duplo lúmen foram os mais utilizados (82,9%). As características sociodemográficas e clínicas da amostra estão apresentadas na Tabela 2.

**Tabela 2:** Características sociodemográficas e clínicas da amostra (n = 35).

**Variável**	**Categoria**	**n (%)**
Sexo	Feminino	21 (60,0%)
	Masculino	14 (40,0%)
Idade	Média ± DP	56,7 ± 13,8 anos
Indicação do acesso venoso	Impossibilidade de acesso periférico	18 (51,4%)
	Quimioterapia	6 (17,1%)
	Cirurgia de grande porte	5 (14,3%)
	Nutrição parenteral total	5 (14,3%)
	Droga vasoativa	1 (2,9%)
Local de punção	Veia subclávia direita	14 (40,0%)
	Veia jugular interna direita	13 (37,1%)
	Veia jugular interna esquerda	4 (11,4%)
	Veia subclávia esquerda	2 (5,7%)
	Veia femoral direita	1 (2,9%)
	Múltiplos sítios	1 (2,9%)
Uso de ultrassonografia	Não	21 (60,0%)
	Sim	14 (40,0%)
Tipo de cateter	Duplo lúmen	29 (82,9%)
	Totalmente implantável	6 (17,1%)

DP = desvio-padrão. Valores expressos em número absoluto e porcentagem, exceto quando indicado.

A Classificação de Campelo et al. distribui as complicações em cinco categorias: I (sem complicações), IIA (complicações menores relacionadas à punção), IIB (complicações menores relacionadas ao posicionamento), IIIA (complicações maiores relacionadas à punção) e IIIB (complicações maiores relacionadas ao posicionamento). Na amostra estudada, a categoria I foi a mais frequente, representando 85,7% dos casos. As complicações menores relacionadas à punção ocorreram em 11,4% dos procedimentos, enquanto as complicações menores relacionadas ao posicionamento foram observadas em 2,9% dos casos. Não foram registradas complicações maiores na amostra estudada.

Entre as complicações menores relacionadas à punção, foram registrados quatro casos, incluindo hematoma local, punção arterial sem repercussão clínica e múltiplas tentativas no mesmo sítio sem necessidade de mudança de local. Nas complicações menores relacionadas ao posicionamento, observou-se um caso de mau posicionamento do cateter sem comprometimento de sua função, com extremidade distal em veia jugular interna.

## DISCUSSÃO

O presente estudo propõe uma classificação objetiva e aplicável para as complicações precoces dos acessos venosos centrais, organizada em cinco categorias e abrangendo eventos relacionados tanto à punção quanto ao posicionamento do cateter. A predominância de procedimentos sem complicações e a ausência de complicações maiores sugerem perfil de segurança favorável no contexto avaliado, embora esse achado deva ser interpretado com cautela em razão do caráter piloto da proposta e do tamanho amostral reduzido.

A principal contribuição da proposta está em sua simplicidade e aplicabilidade prática. Ao padronizar a comunicação sobre complicações em acessos venosos centrais, a classificação pode facilitar a documentação em prontuários, a comparação entre serviços e a elaboração de indicadores assistenciais. Além disso, por abranger tanto eventos relacionados à punção quanto ao posicionamento, oferece uma visão mais ampla do procedimento. Trata-se de um modelo conceitual e operacional, não destinado a funcionar como *checklist* exaustivo nem como instrumento diagnóstico validado.

No cenário brasileiro, as diretrizes da Sociedade Brasileira de Angiologia e de Cirurgia Vascular sobre acesso vascular reforçam o papel da ultrassonografia na orientação da punção, a escolha criteriosa do local de acesso e a confirmação radiológica do posicionamento como medidas associadas à redução de complicações^13^. O alinhamento da classificação proposta a essas recomendações nacionais favorece sua incorporação à prática assistencial no país, na medida em que padroniza a descrição de eventos — relacionados à punção e ao posicionamento — que tais diretrizes buscam prevenir, contribuindo para a construção de indicadores assistenciais comparáveis entre serviços brasileiros.

Entre as limitações do estudo, destacam-se o pequeno tamanho amostral, a realização em centro único, a ausência de complicações maiores e a inexistência de avaliação da reprodutibilidade interobservador. Tais fatores restringem a generalização dos achados e indicam a necessidade de estudos multicêntricos com amostras mais amplas para validação externa da proposta.

## CONCLUSÃO

A classificação proposta é um instrumento piloto para a estratificação de complicações precoces associadas ao acesso venoso central, com potencial de contribuir para a padronização na descrição desses eventos e para a melhora da comunicação entre os profissionais de saúde. Entretanto, as evidências apresentadas neste estudo são limitadas, sobretudo pelo pequeno número de participantes, pelo desenho unicêntrico e pela ausência de complicações maiores na amostra estudada. Portanto, a aplicabilidade e o impacto clínico da classificação ainda necessitam de confirmação em estudos futuros, idealmente com desenho multicêntrico, amostras maiores e avaliação formal da reprodutibilidade interobservador.

## Data Availability

All of the data generated or analyzed are contained in the article and/or supplementary material.
